# BUILDING RESILIENCE IN SKILLED NURSING (BRISK): A CASE OF COMMUNITY-ENGAGED RESEARCH IN SKILLED NURSING FACILITIES

**DOI:** 10.1093/geroni/igad104.1308

**Published:** 2023-12-21

**Authors:** Evan Plys, Christopher Lyons, Ana-Maria Vranceanu

**Affiliations:** Massachusetts General Hospital/ Harvard Medical School, Boston, Massachusetts, United States; Massachusetts General Hospital, Boston, Massachusetts, United States; Massachusetts General Hospital, Boston, Massachusetts, United States

## Abstract

Older adults receiving post-acute rehabilitation care in a skilled nursing facility (SNF) commonly present with high physical, psychological, and social needs. Psychosocial stressors and associated consequences (e.g., poor transitions, re-hospitalizations) may be magnified among families with limited financial resources. The current study aimed to co-develop a dyadic psychological intervention tailored to meet the unique psychosocial needs of socioeconomically disadvantaged patients and their care-partners during the transition from SNF to home. In this presentation, we describe the process of partnering with SNFs and forming a community advisory board (CAB) to meet the objectives of the current study. First, we discuss practical and regulatory considerations related to identifying facility partners (e.g., facility chains, physician groups) and recruiting patient-care-partner dyads in partner facilities. Second, we highlight strategies to incentivize potential SNF partners as well as historically and systematically under-represented patient participants, including budgetary considerations, recruitment strategies, sustained contact after discharge, and clinical service provision. Third, we discuss the recruitment, formation, and role of a CAB with diverse perspectives, including patients, family care-partners, SNF staff, policy makers and advocates. Overall, this presentation will generate discussion on practical strategies for building and maintaining mutually beneficial research partnerships with SNFs and similar hard-to-reach healthcare settings (e.g., long-term care facilities, home health agencies).

